# P53/microRNA-34-induced metabolic regulation: new opportunities in anticancer therapy

**DOI:** 10.1186/1476-4598-13-115

**Published:** 2014-05-21

**Authors:** Ding-Guo Zhang, Jun-Nian Zheng, Dong-Sheng Pei

**Affiliations:** 1 Jiangsu Key Laboratory of Biological Cancer Therapy, Xuzhou Medical College, 84 West Huai-hai Road, 221002 Xuzhou, Jiangsu, China; 2 Center of Clinical Oncology, Affiliated Hospital of Xuzhou Medical College, Xuzhou 221002, China

**Keywords:** LDHA, Metabolism, miR-34, MYC, p53, SIRT1

## Abstract

MicroRNA-34 (miR-34) is directly regulated by p53, and its potential tumor suppressive roles have been studied extensively. As a p53-induced microRNA, miR-34 functions as a tumor suppressor by playing a role in cell cycle arrest, apoptosis and metabolic regulation. Among these p53/miR-34 associated processes, apoptosis and cell cycle arrest are known as essential for p53/miR-34-mediated tumor suppression. P53-mediated metabolic processes have been shown to play pivotal roles in cancer cell biology. Recent studies have also identified several miR-34 targets involved in p53/miR-34-induced metabolic regulation. However, correlations among these metabolic targets remain to be fully elucidated. In this review, we summarize the current progress in the field of metabolic regulation by the p53/miR-34 axis and propose future directions for the development of metabolic approaches in anticancer therapy.

## Introduction

MicroRNAs (miRNAs) are small noncoding RNAs that regulate gene expression by binding to complementary sequences in the 3’UTR of mRNAs. The identification of the first miRNA, Lin-4, which was found in worms [1], was followed by an increasing interest in understanding the realm of small noncoding RNAs. Aberrant regulation of miRNAs is found in many types of human cancer [2,3]. A number of studies have shown that miRNAs can regulate apoptosis, cell proliferation and epithelial-mesenchymal transition in cancer cells [4-7]. Here, we focus on the miR-34 family and investigate the therapeutic potential of p53/miR-34-induced metabolic regulation for the treatment of cancer.

The miR-34 family includes miR-34a, miR-34b and miR-34c, which are encoded by two different genes. MiR-34a is encoded by its own transcript, while miR-34b and miR-34c share a common primary transcript. Members of the miR-34 family have tissue-specific functions, as miR-34a is expressed in the brain, whereas miR-34b/c is largely expressed in the lungs [8,9]. Studies have shown that aberrant expression of the three miR-34 members exists in many cancers [9-16]. MiR-34 genes that are directly regulated by p53 have been identified [8,17,18], and their involvement in p53-mediated cellular responses associated with tumor suppression such as apoptosis [17,19], cell cycle arrest [20,21] and metabolic regulation [22] has been demonstrated. The promotion of apoptosis along with the induction of cell cycle arrest, which are pivotal for p53-mediated tumor suppression, were the first outcomes observed after the ectopic expression of miR-34 [20]. A number of direct miR-34 targets, including B-cell CLL/lymphoma 2 (Bcl-2), baculoviral IAP repeat containing 5 and silent information regulator 1 (SIRT1), are related to cellular apoptosis [23]. Another class of miR-34 targets including v-myc avian myelocytomatosis viral oncogene homolog (MYC), E2F transcription factor 3 and cyclin E2 are involved in the induction of G1 arrest [10,11,24], and miR-34 targets associated with metabolic regulation governed by p53 have also been identified [22,25]. P53/miR-34-mediated apoptosis and cell cycle arrest have been studied extensively, whereas metabolic regulation through the p53/miR-34 axis is not fully understood, in particular the correlations among miR-34-induced metabolic targets. Recently, an increasing number of studies have shown an association between metabolism and p53, which indicates that metabolic regulation may play a critical role in p53-induced tumor suppression and suggests that the roles of metabolism and canonical processes of p53 in human cancer need further evaluation [26,27]. In addition, miR-34 has been implicated in metabolism and metabolic disorders [28-30]. In the present review, we summarize the evidence and research progress in metabolic regulation mediated by p53/miR-34 to shed light on the potential of the p53/miR-34 axis as a therapeutic target for the treatment of cancer.

## Tumor suppressive roles of p53-mediated metabolism

Studies have shown that the metabolic targets of p53 are associated with glycolysis [31-34], mitochondrial respiration [35] and lipid metabolism [36]. Regarding p53 function, cell cycle arrest and apoptosis have been studied extensively and recognized as essential for p53-induced tumor suppression. However, recent studies found that these canonical processes are not the rate-limiting steps in tumor suppression and suggested metabolic regulation as a crucial step for early-onset spontaneous tumorigenesis [26,27]. They generated mice bearing lysine to arginine mutations at one (p53^K117R^) or three (p53^3KR^; K117R + K161R + K162R) p53 acetylation sites and found that p53-mediated cell cycle arrest and apoptosis were abrogated by the loss of acetylation at all three sites. Interestingly, early-onset tumor formation did not occur in animals carrying either mutation, suggesting that the combined loss of p53-dependent cell cycle arrest, apoptosis and senescence was inadequate to abolish the p53-mediated tumor suppression. The fact that p53^3KR^ retains the ability to regulate the expression of metabolic p53 target genes, together with data on glucose uptake and glycolysis tests, led the authors to suggest that p53^3KR^ retains its activity at least partly through the regulation of energy metabolism levels in vivo. Further evidence was provided by additional studies in which they generated mice deficient in the p53 proapoptotic effectors p21, Puma and Noxa [27], the expression of which was only reduced rather than abrogated in the former study. This study also confirmed that instead of cell cycle arrest and apoptosis, metabolic regulation might be critical for p53-mediated tumor suppression. These results provided a new angle to further investigate the mechanism underlying the function of p53 in tumor suppression.

## Feedback loops in the p53/microRNA-34 network

In 2007, miR-34 family members were reported to be direct p53 targets and shown to induce apoptosis and cell cycle arrest [17,18]. Further studies indicated that miR-34 functions as a tumor suppressive miRNA and plays a role in the regulation of p53 expression, and in turn p53 directly regulates miR-34 and thereby induces cellular processes associated with tumor suppression. MiR-34 can repress SIRT1 [30], histone deacetylase 1 (HDAC1) [22,37] and the transcriptional factor YY1 [22,38,39], and thereby forms positive feedback loops for p53 activation. An increasing number of miR-34 targets have been identified and many of them are associated with tumor suppression, with most of them governed by p53. P53/miR-34-mediated tumor suppressive processes include apoptosis and cell cycle arrest, stemness, metastasis and metabolism [40-43]. Among these cellular responses, apoptosis and G1 arrest were the first processes associated with ectopic expression of miR-34 and have been studied extensively. However, other p53/miR-34-induced processes such as metabolic regulation remain to be fully elucidated.

## Collaboration of p53/microRNA-34 targets in metabolic regulation

Several miR-34 targets are associated with metabolic processes, among which lactate dehydrogenase A (LDHA), MYC and SIRT1 are of central importance [10,22,25,44]. These three direct miR-34 targets have tight connections with one another and function collaboratively in p53/miR-34-mediated metabolic regulation.

Numerous tumor suppressors and oncogenes are closely associated in metabolic pathways, and the first documented mechanism involving an activated oncogene in altered glucose metabolism was the transcriptional activation of LDHA by the oncogenic transcription factor MYC [45]. LDHA catalyzes the conversion of pyruvate to lactate and is considered to play a key role in anaerobic glycolysis [46]. The level of LDHA is elevated in many human cancers in correlation with tumor proliferation and malignant growth [47,48]. MYC is frequently altered in human cancer and has been reported to regulate many glucose metabolism genes, such as glucose transporter GLUT1 and hexokinase 2 (HK2) [49]. The regulation of cell size, proliferation and cellular metabolism by MYC was proposed to occur in a miR-34-dependent manner [10,49,50]. MYC was shown to transactivate the LDHA promoter and directly increase LDHA expression, which suggested that LDHA is a direct target of MYC. Furthermore, the elevated expression of LDHA may be necessary for MYC-mediated transformation [45]. LDHA was also shown to be a common target of MYC and the hypoxia inducible factors (HIFs) [51]. HIFs, as well as MYC, are pivotal factors for tumorigenesis in many types of human cancers and are able to activate LDHA [52,53]. The ectopic expression of MYC promotes its collaboration with HIF to confer metabolic advantages to tumor cells, which partly contributes to the Warburg effect [51,54]. In this process, the expression of LDHA is activated by both MYC and HIFs. Further studies showed that alterations of LDHA expression lead to changes in cancer metabolism [47,55-57].

Another miR-34 target, SIRT1, also plays a critical role in p53/miR-34-induced metabolism. SIRT1 is a multifaceted NAD^+^-dependent protein deacetylase that deacetylates p53, resulting in the inhibition of its activity [58]. MiR-34 inhibits SIRT1 expression, resulting in a reduction of p53 deacetylation [25]. Thus, SIRT1, p53 and miR-34 form a positive feedback loop to induce tumor suppression. Additionally, SIRT1 can inhibit the miR-34 promoter through histone deacetylation [59].

Close associations between SIRT1 and LDHA have been described, and SIRT1 activity is largely determined by LDHA. SIRT1 is a NAD^+^-dependent protein deacetylase. NAD^+^ is regenerated from NADH through the reduction of pyruvate to lactate. LDHA plays an essential part in the metabolism of pyruvate and NADH, and thus sustains cellular NAD^+^ levels [47,60,61]. Therefore, SIRT1 is under strict control of another miR-34 target, LDHA. Moreover, studies investigating the effect of SIRT1 on LDHA found that depletion of SIRT1 resulted in reduced transcription of LDHA [62].

The correlation between SIRT1 and MYC has also been investigated. A recent study identified a positive feedback loop consisting of MYC, the nicotinamide-phosphoribosyltransferase (NAMPT) enzyme, the SIRT1 inhibitor deleted in breast cancer 1 (DBC1) and SIRT1 [63]. This study showed that MYC activates SIRT1, which in return promotes MYC function. MYC induces SIRT1 deacetylase activity and the MYC-induced NAMPT mediates the activity of SIRT1, since the NAD+/NADH ratio is crucial for SIRT1. Moreover, MYC may also activate SIRT1 by directly binding to the SIRT1 inhibitor DBC1. Two separate mechanisms contributing to the MYC-induced SIRT1 activation were identified: 1. MYC induces NAMPT, resulting in an increase in the SIRT1 cofactor NAD+. 2. The MYC-DBC1 association facilitates SIRT1 activation. Reciprocally, these authors found that SIRT1-mediated deacetylation increases the half-life of MYC, and SIRT1 increases MYC transcriptional activity. Although certain molecular details remain unclear, such as the mechanisms of SIRT1 stabilization and DBC1-SIRT1 interplay, these data highlighted the clinical potential of targeting this feedback loop. Another study showed the positive effect of SIRT1 on MYC by demonstrating that the SIRT1-mediated deacetylation of MYC could promote the association with its essential partner MYC associated factor X, thereby promoting the transcriptional activity of MYC [62].

A possible positive feedback loop consisting of LDHA, MYC and SIRT1 may function collaboratively to regulate cellular metabolism via the p53/miR-34 axis. LDHA, MYC and SIRT1 are direct miR-34 targets and are involved in the p53-mediated metabolic regulation. Rather than functioning independently as miR-34 targets, SIRT1, MYC and LDHA work cooperatively in p53/miR-34-mediated metabolic regulation, thereby acting in a collaborative form instead of in parallel.

Several other p53-induced miRNAs, such as let-7, miR-107 and miR-200, may function as direct and indirect regulators of LDHA, MYC and SIRT1 and may broaden our view of the collaborations between miR-34 and other p53-induced miRNAs in metabolic regulation.

**Figure 1:**
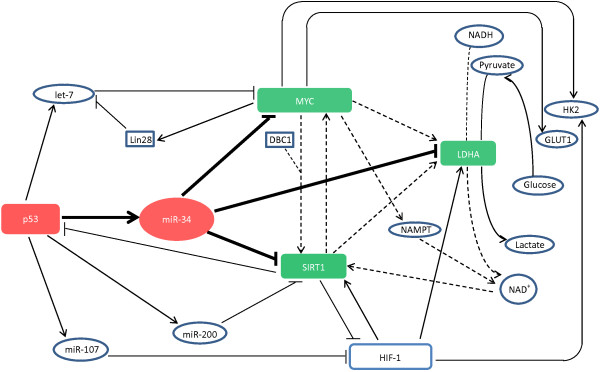
**Metabolic regulation by the p53/miR-34 axis and contribution of other related p53-induced miRNAs to the regulatory network.** The involvement of LDHA, MYC and SIRT1 in the metabolic regulatory network is illustrated (dotted lines), and the importance of their interaction for miR-34-induced metabolic activity governed by p53 is suggested in the diagram.

The Let-7 miRNA family is one of the most conserved and ancient miRNAs and was found to be regulated by p53 [64,65]. The Lin28/let-7 axis regulates glucose metabolism and the RNA-binding protein Lin28 can promote malignancy through the selective inhibition of let-7 biogenesis [66,67]. MYC binds to the Lin28 promoter and regulates its expression. Studies have found that Lin28b plays an essential role in MYC-induced let-7 repression and the MYC/let-7/Lin28 pathway is pivotal for cellular transformation [66]. Although further research is needed, it is possible that the let-7-MYC/Lin28 axis plays important roles in glycolytic regulation and this could subsequently contribute to a more complex network involved in p53/miR-34-mediated metabolic regulation. MiR-107 is regulated by p53 and is encoded within an intron of the p53-induced pantothenate kinase 1 gene, PANK1 [68]. Studies have found that p53-induced miR-107 can decrease HIF-1β expression, thereby suppressing the transcriptional response to hypoxia and inhibiting tumor angiogenesis [69]. HIF-1β is a subunit of HIF-1 that can modulate hypoxic responses without affecting HIF-1α [69]. Since HIF-1 is a regulator of the miR-34 target LDHA, p53-induced miR-107 activation may suppress LDHA expression and lead to the suppression of glycolysis. In addition to LDHA and MYC, p53/miR-107-induced HIF-1 suppression is related to another miR-34 target, SIRT1. SIRT1 negatively regulates HIF-1 through direct deacetylation, and conversely, HIF-1 promotes SIRT1 transcription [70,71]. Thus, the miR-107 target HIF-1 has tight connections with LDHA, SIRT1 and MYC. Moreover, miR-200 was also reported to regulate SIRT1 expression [72]. In summary, the collaborations among LDHA, MYC and SIRT1 can indeed widen the window of miR-34-induced tumor suppression (Figure 1).

## Therapeutic potential of p53/miR-34-induced metabolic targets

Since p53 is mutated in approximately 50% of human cancers and miR-34 is a critical component of p53 signaling pathways, the p53/miR-34-induced metabolic targets may have therapeutic potential. LDHA is a common target of MYC and HIF, both of which work collaboratively in the Warburg effect and are crucial for cellular metabolism. Moreover, NADH-dependent LDHA regenerates NAD + and competes with the mitochondrial NADH/NAD + shuttle system, which is decisive for mitochondrial activity associated with tumor metabolism [73]. Inhibition of LDHA can limit the energy supply and consequently suppress the metastatic and invasive potentials of tumor cells [46,74]. This has increased interest in the therapeutic potential of LDHA as a novel target for tumor suppression. Studies have found that total deficiency of LDHA is not associated with any specific symptoms under normal conditions, which indicates that inhibition of LDHA activity could be a nontoxic therapeutic approach to induce tumor suppression [48]. Furthermore, a number of studies have shown that LDHA levels are associated with tumor sensitivity and resistance to therapeutic agents [46,74,75]. LDHA has also been suggested as a potential target for chemotherapies. SIRT1 and MYC are also associated with cellular apoptosis and cell cycle arrest through the p53/miR-34 axis [10,25]. The development of small-molecule inhibitors of SIRT1 for the treatment of cancer is under consideration, and activation of SIRT1 alone was suggested as sufficient to induce tumor suppression in human cancers with mutated p53 [76-78]. Moreover, increased levels of MYC are detected in a large number of human cancers. After the first established link between MYC and LDHA, many other glucose metabolism genes have been shown to be governed by MYC, such as glucose transporter 1 and hexokinase 2 [79,80]. Therefore, understanding the therapeutic effects of targeting multiple MYC-mediated metabolic pathways may be crucial for the treatment of cancer.

Several as yet unidentified mechanisms may play a role in the loop consisting of SIRT1, LDHA and MYC, and they should be considered when it comes to therapeutic use. For instance, how MYC overexpression works to regulate both apoptosis and cellular metabolic state remains an unanswered question [51]. Moreover, the potential clinical use of SIRT1 can be complicated: SIRT1 overexpression is oncogenic in wild-type p53, whereas it plays a tumor-suppressive role in cells with mutated p53. Although this phenomenon has been proposed to be related to p53 status [78], its underlying mechanism requires further clarification. In addition, the exact mechanism of how SIRT1 induces metabolic regulation remains unknown. These uncertainties in the roles of miR-34-induced metabolic regulation need to be clarified.

## Conclusions

In this review, we described the functions of three direct miR-34 targets and their collaboration in regulating metabolism via the p53/miR-34 axis, as well as additional p53-induced miRNAs with the same metabolic targets as miR-34. Additionally, we discussed the significance of these miR-34-induced metabolic targets for anticancer therapies. In summary, metabolic regulation via the p53/miR-34 axis may be crucial for tumor suppression and therefore, the development of small-molecule drugs targeting LDHA, SIRT1 and MYC may be a novel strategy for anticancer therapy.

## Abbreviations

miRNA: microRNA; miR-34: microRNA-34; miR-107: microRNA-107; miR-200: microRNA-200; HIF-1: Hypoxia inducible factor-1; LDHA: Lactate dehydrogenase A; SIRT1: Sirtuin 1.

## Competing interests

The authors declare that they have no competing interests.

## Authors’ contributions

DGZ collected and read the related paper and drafted the manuscript. DSP and JNZ participated in the design of the review and helped to draft the manuscript. All authors read and approved the final manuscript.
